# The diagnostic and prognostic role of cerebrospinal fluid biomarkers in glucose transporter 1 deficiency: a systematic review

**DOI:** 10.1007/s00431-024-05657-6

**Published:** 2024-07-02

**Authors:** Mario Mastrangelo, Filippo Manti, Giacomina Ricciardi, Elisa Maria Colacino Cinnante, Noemi Cameli, Annachiara Beatrice, Manuela Tolve, Francesco Pisani

**Affiliations:** 1 Woman/Child Health and Urological Sciences Department, Sapienza University of Rome, Via dei Sabelli 108, 00185 Rome, Italy; 2 Unit of Child Neurology and Psychiatry, Department of Neuroscience/Mental Health, Azienda Ospedaliero Universitaria Policlinico Umberto, Rome, Italy; 3 Department of Human Neuroscience, Sapienza University of Rome, Rome, Italy; 4 Clinical Pathology Unit, Azienda Ospedaliero-Universitaria Policlinico Umberto I, Rome, Italy

**Keywords:** Epilepsy, Movement disorders, Genetic epilepsies, Neurodevelopmental disorders, Children

## Abstract

The purpose of this study is to investigate the diagnostic and prognostic role of cerebrospinal fluid (CSF) biomarkers in the diagnostic work-up of glucose transporter 1 (GLUT1) deficiency. Reported here is a systematic review according to PRISMA guidelines collecting clinical and biochemical data about all published patients who underwent CSF analysis. Clinical phenotypes were compared between groups defined by the levels of CSF glucose (≤ 2.2 mmol/L versus > 2.2 mmol/L), CSF/blood glucose ratio (≤ 0.45 versus > 0.45), and CSF lactate (≤ 1 mmol/L versus > 1 mmol/L). Five hundred sixty-two patients fulfilled the inclusion criteria with a mean age at the diagnosis of 8.6 ± 6.7 years. Patients with CSF glucose ≤ 2.2 mmol/L and CSF/blood glucose ratio ≤ 0.45 presented with an earlier onset of symptoms (16.4 ± 22.0 versus 54.4 ± 45.9 months, *p* < 0.01; 15.7 ± 23.8 versus 40.9 ± 38.0 months, *p* < 0.01) and received an earlier molecular genetic confirmation (92.1 ± 72.8 versus 157.1 ± 106.2 months, *p* < 0.01). CSF glucose ≤ 2.2 mmol/L was consistently associated with response to ketogenic diet (*p* = 0.018) and antiseizure medications (*p* = 0.025). CSF/blood glucose ratio ≤ 0.45 was significantly associated with absence seizures (*p* = 0.048), paroxysmal exercise‐induced dyskinesia (*p* = 0.046), and intellectual disability (*p* = 0.016) while CSF lactate > 1 mmol/L was associated with a response to antiseizure medications (*p* = 0.026) but not to ketogenic diet.

*Conclusions*:This systematic review supported the diagnostic usefulness of lumbar puncture for the early identification of patients with GLUT1 deficiency responsive to treatments especially if they present with co-occurring epilepsy, movement, and neurodevelopmental disorders.

**Table:** 

**What is Known:**
*• Phenotypes of GLUT1 deficiency syndrome range between early epileptic and developmental encephalopathy to paroxysmal movement disorders and developmental impairment*
**What is New:**
*• CSF blood/glucose ratio may predict better than CSF glucose the diagnosis in children presenting with early onset absences*
*• CSF blood/glucose ratio may predict better than CSF glucose the diagnosis in children presenting with paroxysmal exercise induced dyskinesia and intellectual disability.*
*• CSF glucose may predict better than CSF blood/glucose and lactate the response to ketogenic diet and antiseizure medications.*

**Supplementary Information:**

The online version contains supplementary material available at 10.1007/s00431-024-05657-6.

## Introduction

Glucose transporter 1 (GLUT1) deficiency syndrome is a rare and treatable neurometabolic disorder with a multifaceted phenotypic spectrum ranging between early onset epileptic and developmental encephalopathy, early onset absence or myoclonic-atonic epilepsy, focal epilepsy, episodic choreoathetosis and spasticity, paroxysmal exercise-induced dyskinesia, intermittent ataxia, and various degrees of neurodevelopmental impairment [1, 2]. These presentations are caused by pathogenic variants of solute carrier family 2 member 1 (SLC2A1; OMIM 138140) gene encoding for the most important energy carrier of the brain across the blood-brain barrier (GLUT1) [1].

The early diagnosis of GLUT1 deficiency has remarkable prognostic implications because an effective treatment (e.g., ketogenic diet) may result in a complete resolution of motor symptoms and epileptic seizures [3]. Lumbar puncture has always represented the initial diagnostic step since the first descriptions of GLUT1 deficiency even if the increasing potentials of ultra-fast next-generation sequencing techniques, the recent validation of a less invasive blood test (e.g., METAGLUT1), and the detection of novel candidate cerebrospinal biomarkers (e.g., gluconic + galactonic acid, xylose-α1-3-glucose, and xylose-α1-3-xylose-α1-3-glucose) provided possible alternative diagnostic tools [4, 5].

The precise definition of the diagnostic strength and the prognostic role of each measurable CSF biomarker in the real-world practice still represent an important gap in the literature. This review aimed to evaluate whether CSF glucose, CSF/blood glucose ratio, and CSF lactate may impact the phenotyping process of patients with GLUT1 deficiency and guide therapeutic choices.

## Materials and methods

**Fig. 1:**
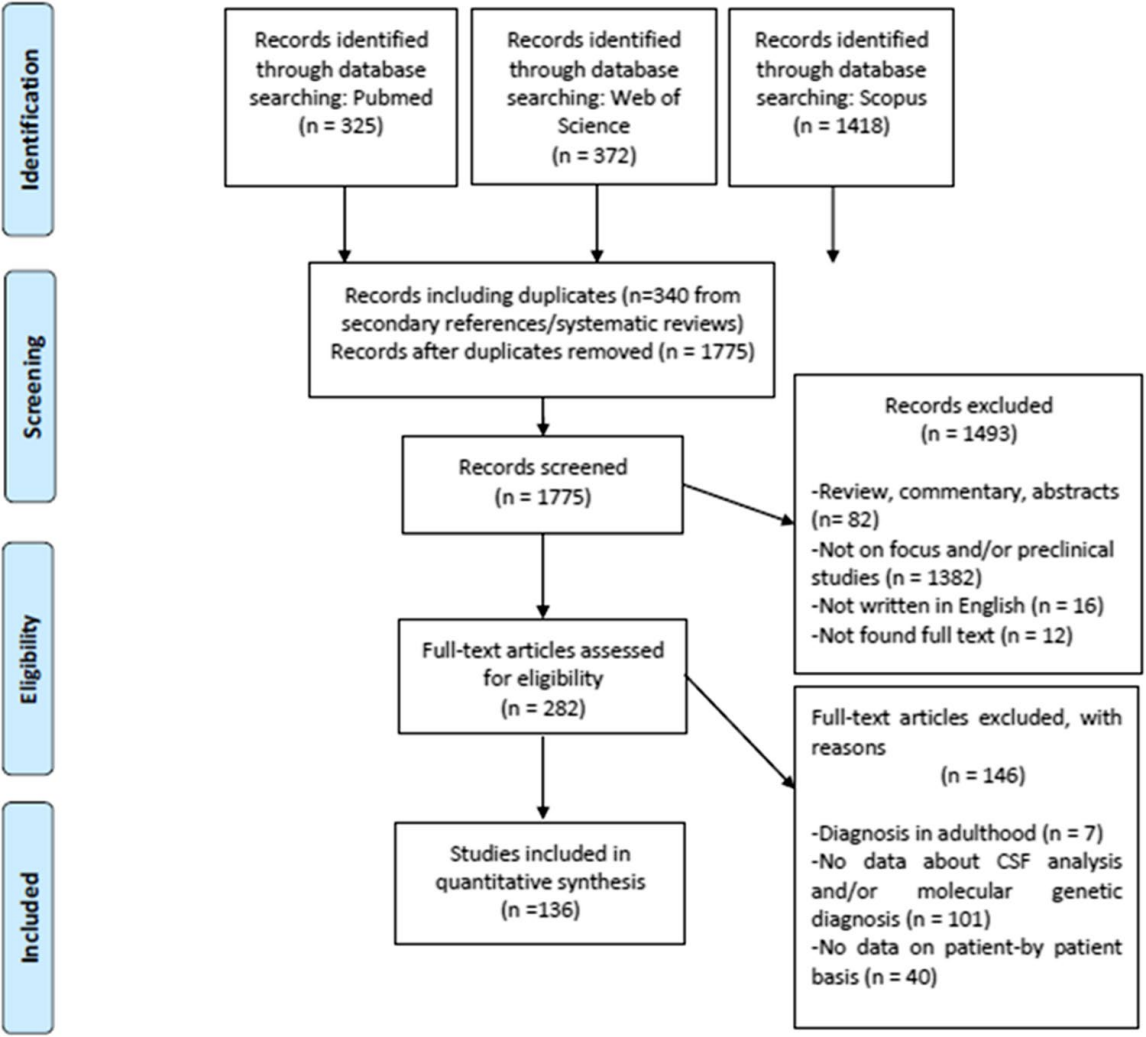
Flow-chart illustrating the selection of the articles analyzed in the present systematic review according to PRISMA guidelines

We conducted a systematic review of published pediatric cases with a compatible clinical phenotype and a confirmed molecular genetic diagnosis of GLUT1 deficiency syndrome according to PRISMA guidelines (Fig. 1). A PubMed, Web of Science, and Scopus search was performed using the search terms (GLUT1 OR “glut 1” OR glut-1 OR “glucose transporter type 1”) AND (deficit* OR disorder) and filtered results for the age range 0–18 years and the temporal range January 1991–June 2023. Reference lists of each selected article and systematic reviews on the same focus were reviewed to collect additional papers.

Articles reporting patients without data about CSF analysis or without a molecular genetic diagnosis, articles without data reported on patient-by-patient basis, articles about studies not focusing on human beings, and articles that were not written in English were excluded from the analysis (Fig. 1).

Results were screened by title, abstract, and full text. Duplicates were excluded. Studies reporting overlapping cohorts were identified by comparing relevant features (i.e., number of subjects, time variables, outcomes, institution, and year). The studies with the most extensive reporting were selected for inclusion in our analysis.

Data on demographic features, epilepsy, movement disorders and developmental phenotypes, CSF biochemical markers, molecular genetic data, and neurological outcomes were collected for each of the patients satisfying the inclusion criteria.

Figure 1 summarizes the features of the selected articles. Study selection was performed independently by three authors (ECC, NC, and AB). Supplement 1 includes the complete data collection sheet including the main demographic, clinical, and biochemical data for each one of the published patients. Supplement 2 summarizes the distribution of the associated SLC2A1 gene variants. Supplement 3 illustrates the evaluation of the articles that was realized according to NIH-Quality Assessment Tool (https://www.nhlbi.nih.gov/health-topics/study-quality-assessment-tools). Supplement 4 and 5 report the risk of bias for each one of the selected article according to the RoB 2.0-Robvis tool (https://mcguinlu.shinyapps.io/robvis/). Supplement 6 includes PRISMA checklist.

Clinical data were compared between (1) patients with CSF glucose ≤ 2.2 and patients with CSF glucose > 2.2 mmol/L [2], (2) patients with CSF/blood glucose ratio ≤ 0.45 and patients with CSF/blood glucose ratio > 0.45 [2], and (3) patients with CSF lactate < 1 mmol/L and patients with CSF lactate > 1 mmol/L (Table 1). Cut-off values for CSF glucose and CSF /blood glucose ratio were taken from the literature [2]. Cut-off values of CSF glucose, CSF /blood glucose ratio, and CSF lactate were also calculated through receiver operating characteristic (ROC) curve analysis (sensitivity, specificity, and Youden index) within the cohort of published patients reported here.

**Table 1:** Relationships between demographic, neurological, and neurodevelopmental features and CSF biomarkers

Variables	CSF glucose ≤ 2.2 mmol/L	CSF glucose < 2.2 mmol/L	CSF glucose > 2.2 mmol/L	CSF/blood glucose ratio ≤ 0.45	CSF/blood glucose ratio < 0.45	CSF/blood glucose ratio > 0.45	CSF lactate ≤ 1 mmol/L	CSF lactate < 1 mmol/L	CSF lactate > 1 mmol/L	*X*^2^/*p*
Demographic data	**Number of patients**	*N* = 376	*N* = 55	-	*N* = 407	*N* = 78	-	N = 98	*N* = 79	-
	**Sex**	166M+155F+55?	32M+18F+5?	0.105	170M+ 185F +52?	47M+27F+4?	0.015*	42M+44F+11?	38M+41F	1
	**Age at the onset of symptoms (months, M ± SD)**	16.4±22.0	54.4±45.9	<0.01**	15.7±23.8	40.9±38.0	<0.01**	17.2±27.2	18.05±24.1	0.160
	**Age at molecular genetics diagnosis (months, M ± SD)**	92.1±72.8	157.1±106.2	<0.01**	100.1±84.0	120.7±75.6	0.039*	97.9± 71.5	112.2± 74.6	0.376
Seizure types	**Absence**	*N* = 82 (29M+49F+4?)	*N* = 23 (12M+9F+2?)	0.135	*N* = 104 (33M+ 61F +10?)	*N* = 30 (16M+ 12F+2?)	0.048*	*N* = 21 (5M+ 12F+4?)	*N* = 16 (4M+ 12F)	1
	**Tonic-clonic**	*N* = 71 (32M+38F+1?)	*N* = 9 (6M+3F)	0.299	*N* = 79 (29M+ 49F+1?)	*N* = 21 (12M+ 9F)	0.135	*N* = 11 (5M+ 6F)	*N* = 6 (3M+3F)	1
	**Myoclonic**	*N* = 71 (33M+34F+4?)	*N* = 2M	0.493	*N* = 76 (34M+39F+3?)	*N* = 7 (4M+3F)	0.702	*N* = 13 (6M+ 6F+1?)	*N* = 13 (4M+ 9F)	0.428
Movement disorder types	**Ataxia**	*N* = 203 (87M+97F +19?)	*N* = 8 (6M+1F+1?)	0.059	*N* = 204(100M +100F+3?)	*N* = 16 (9M +6F+1?)	0.594	*N* = 61 (28M +31F+2?)	*N* = 42 (24M +18F)	0.419
	**Dystonia**	*N* = 85 (38M+35F +12?)	*N* = 15 (12M+3F)	0.084	*N* = 87 (48M+ 36F+3?)	*N* = 15 (11M+4F)	0.270	*N* = 31 (17M +13F+1?)	*N* = 11 (4M+ 6F+1?)	0.473
	**PED**	*N* = 51 (20M+22F+11?)	*N* = 18 (12M+5F+1?)	0.151	*N* = 57 (20M+ 31F+6?)	*N* = 25 (16M+ 8F+1?)	0.046*	*N* = 16 (3M+ 9F+4?)	*N* = 11 (4M+ 6F+1?)	0.652
	**Chorea/choreoatetosis**	*N* = 19 (12M+7F)	*N* = 7 (5M+2F)	1	*N* = 16 (9M+ 7F)	*N* = 15 (11M+ 4F)	0.458	*N* = 5 (3M+ 2F)	*N* = 3 (2M+ 1F)	1
	**Cerebral palsy**	*N* = 14 (9M+5F)	*N* = 1F	0.400	*N* = 22 (10M+ 12F)	*N* = 3M	0.220	*N* = 5 (4M+1F)	*N* = 5 (1M+ 4F)	0.206
Neurodevelopmental disorders and cognitive profiles	**Language disorder**	*N* = 22 (13M+9F)	*N* = 1M	0.181	*N* = 23 (14M +8F+1?)	*N* = 4 (1M+3F)	0.279	*N* = 8 (5M+ 2F+1?)	*N* = 5 (4M+ 1F)	1
	**GDD**	*N* = 66 (23M+30F +13?)	*N* = 2M	0.202	*N* = 60 (25M+ 27F+8?)	*N* = 4 (2M+ 2F)	1	N = 19 (8M+ 5F+6?)	*N* = 20 (7M+ 13F)	0.169
	**Learning disability**	*N* = 23 (14M+9F)	*N* = 8 (3M+4F+1?)	0.665	*N* = 5 (2M+3F)	*N* = 2 (1M+ 1F)	1	*N* = 13 (5M+ 8F)	*N* = 6 (5M+1F)	0.141
	**IQ in the borderline range**	*N* = 11 (6M+5F)	*N* = 4 (3M+1F)	0.604	*N* = 13 (4M+6F+3?)	*N* = 8 (5M+ 3F)	0.637	*N* = 1M	*N* = 2F	-
	**ID**	*N* = 154 (74M+73F +7?)	*N* = 16 (10M+6F)	0.434	*N* = 212 (92M+ 102F+18?)	*N* = 24 (18M+ 6F)	0.016*	*N* = 46 (20M +25F+1?)	*N* = 42 (21M +21F)	0.670
	Mild ID	*N* = 69 (28M+40F +1?)	*N* = 9 (6M+3F)	0.172	*N* = 91 (33M+ 54F+4?)	*N* = 12 (8M+ 4F)	0.068	*N* = 21 (11M +10F)	*N* = 20 (9M+ 11F)	0.758
	Moderate ID	*N* = 36 (21M+15F)	*N* = 2 (1M+1F)	1	*N* = 48 (25M+ 18F+5?)	*N* = 3M	0.269	*N* = 8 (3M+ 5F)	*N* = 6 (4M+ 2F)	0.592
	Severe ID	*N* = 21 (13M+8F)	*N* = 1M	1	*N* = 45(22M+ 19F+4?)	*N* = 5M	0.247	*N* = 7 (3M+ 4F)	*N* = 5 (4M+ 1F)	0.293
	ID unspecified	*N* = 28 (12M+10F+6?)	*N* = 4 (2M+2F)	1	*N* = 28 (12M+ 11F+5?)	*N* = 4 (2M+ 2F)	1	*N* = 10 (4M+5F+1?)	*N* = 11 (4M+7F)	1
	**ADHD**	*N* = 5 (4M+1F)	-	-	*N* = 3M	*N* = 3 (1M+ 2F)	1	-	*N* = 1M	-
Efficacy of treatment	**Ketogenic diet**	*N* = 180 (88M+ 92F)	*N* = 13 (11M+2F)	0.018*	*N* = 196 (93M +96F+10?)	*N* = 24 (12M+ 12F)	1	*N* = 55 (26M +29F)	*N* = 45 (22M +23F)	1
	**Antiseizure medications**	*N* = 38 (17M+21F)	*N* = 9 (8M+1F)	0.025*	*N* = 31 (12M +19F)	*N* = 14 (10M+ 4F)	0.057	*N* = 8 (6M+2F)	*N* = 14 (3M+ 11F)	0.026*
	**Ketogenic diet + antiseizure medications**	*N* = 26 (7M+19F)	*N* = 6 (4M+2F)	0.147	*N* = 24 (7M+ 17F)	*N* = 8 (4M+4F)	0.397	*N* = 5 (3M+2F)	*N* = 11 (2M+ 9F)	0.244

*M* mean, *SD* standard deviation, *PED* paroxysmal exercise‐induced dyskinesia, *GDD* global developmental delay, *ID* intellectual disability, *ADHD* attention deficit hyperactivity disorder

^*^p < 0.05; **p < 0.01

Data extraction from all the selected articles was blindly performed by two authors (FM and GR). Controversies about study selection and data extraction were solved after a case-by-case discussion involving all the authors with the supervision of FP as Senior Author.

All statistical analyses were conducted using IBM SPSS Statistics version 25.0 (SPSS Inc., Chicago, IL, USA). Normality was assessed with the Kolmogorov-Smirnov test. Comparisons were performed using Mann–Whitney *U* test for non-normally distributed data and Fisher’s exact test for categorical variables. A *p* value < 0.05 represented statistical significance for all tests. Pairwise deletion was applied for each one of the analyzed parameters. Variables with missing data above a certain threshold (e.g., 20%) were scrutinized to decide on their processing.

The review was recorded in PROSPERO (https://www.crd.york.ac.uk/prospero/-CRD42023480301) on 7/11/2023, and the registered protocol was updated on 3/5/2024 to include an extension of the initially planned completion date.

## Results

### Patient characteristics

The screening of the literature resulted in 136 articles fulfilling all the inclusion criteria (60 case reports, 47 case series, 15 retrospective cohort studies, 9 prospective cohort studies, 4 case-control studies, and 1 survey). The quality of the article according to NIH-Quality Assessment Tool was judged as fair in 78, good in 44, and poor in 17 cases (Suppl. 3). A consistent risk of bias was assessed by RoB 2.0-Robvis tool (https://mcguinlu.shinyapps.io/robvis/) especially for the studies focused on the measurement of outcome.

Our search strategy yielded 562 patients with a confirmed genetic diagnosis of GLUT1 deficiency syndrome with a male/female ratio of 1.06 and a mean age at the diagnosis of 8.7 ± 6.7 years (range, 22 days–39 years; data available for 279 patients) (Fig. 1, Suppl. 1) [2, 3, 5–140].

Lumbar puncture was mostly performed before the molecular-genetic confirm (Suppl. 1).

Epileptic seizures were reported in 457 patients (Suppl. 1). Predominant seizure types at the onset mainly included absences (151 patients), myoclonic (95 patients), and generalized tonic-clonic seizures (97 patients) while focal onset was observed in 70 patients (Suppl. 1). EEG features were reported in 244 patients. The most frequent EEG patterns included diffuse spike and wave discharges (127 patients) with a frequency of 3–4 Hz being reported in 17 patients and a frequency of 1.5–2.5 Hz in 15 patients (Suppl. 1). Focal epileptiform abnormalities and slow abnormalities were respectively detected in 47 and 55 patients (Suppl. 1).

Data about the administered antiseizure medications were available for 208 published patients while details about the related therapeutic response were provided in 118 cases (seizures reduction reported in 53) (Suppl. 1). A remarkable proportion of them had received GLUT1 inhibitors such as valproate (95 patients), benzodiazepines (9 patients), phenobarbital (19 patients), or phenytoin (11 patients) before the diagnosis (Suppl. 1) with an apparently paradoxical efficacy in 26 patients.

Four hundred forty-eight patients presented with movement disorders mainly including intermittent or persistent ataxia (262 patients), dystonia (190 patients), paroxysmal exercise-induced dyskinesia (81 patients), and different pyramidal signs (78 patients) (Suppl. 1).

A neurodevelopmental disorder was diagnosed in 404 patients (Suppl. 1). An intellectual disability was assessed in 247 patients (25 patients had a borderline intelligence quotient (IQ) while different degrees of delay in the achievement of developmental milestones were reported in 78 patients (Suppl. 1) with the predominant involvement of language domains in 29 patients and of learning in other 31 cases (Suppl. 1)).

Details about the administration of ketogenic diet were reported for 379 patients; data about the response was available for 263 patients and 234 of them experienced a clinical improvement of epilepsy and/or movement disorders (Suppl. 1).

The duration of follow-up was reported for 89 patients (mean duration = 3.05 ± 3.95 years) (Suppl. 1). Data about long-term outcome were reported for a proportion of patients ranging between 23.9 and 37.9% (213 about epilepsy, 149 about movement disorders, and 135 about neurodevelopmental disorders) (Suppl. 1). A reduction/disappearance of seizures was reported in 158 patients while motor symptoms improved in 116 patients and cognitive/developmental gains occurred in 91 (Suppl. 1).

Published genotypes included 21 variants carried by 55.2% of patients (Supplement 2). Most commonly reported variants included c.997C>T (p.Arg333Trp), c.376C>T (p.Arg126Cys), c.283_284delinsAT (p.Ser95Ile), c.377G>A (p.Arg126His), and c.884C>T (p.Thr295Met). Most of the variants were scarcely correlated with the values of CSF biomarkers with a distribution covering all possible biochemical phenotypes. c.997C>T (p.Arg333Trp), c.457C>T (p.Arg153Cys), and c.884C>T (p.Thr295Met) were predominantly observed in patients with CSF glucose ≤ 2.2 mmol/L and in patients with CSF/blood glucose ratio ≤ 0.45 (Supplement 1). Conversely, c.283_284delinsAT (p.Ser95Ile) was mainly observed in patients with CSF glucose > 2.2 mmol/L (Supplement 1).

### CSF biomarkers and associated phenotypes

**Fig. 2:**
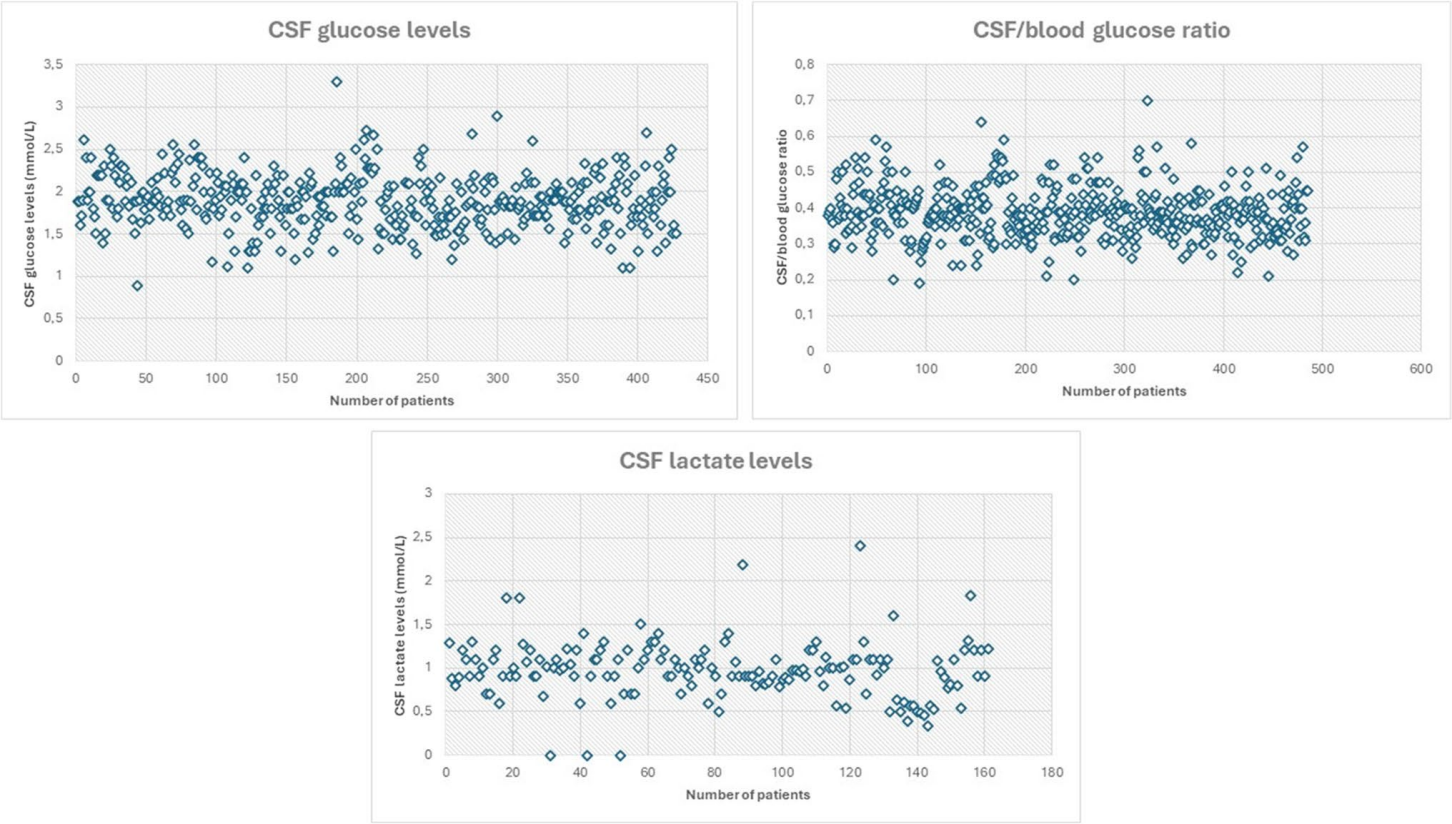
Distribution of CSF glucose, CSF/blood glucose ratio, and CSF lactate levels in the 537 patients included in the analysis of this systematic review

Table 1 and Figure 2 summarize the available data about levels of CSF biomarkers and associated phenotypes.

ROC analysis evidenced that CSF glucose had a sensitivity of 87%, a specificity of 100%, and a Youden index of 87% at 2.2 mmol/L. CSF/blood glucose ratio showed a sensitivity of 87%, a specificity of 100%, and a Youden index of 87% at values > 0.45. Sensitivity, specificity, and Youden index for CSF lactate at 1.0 ng/mL were 95%, 98%, and 93%, respectively.

Patients with CSF glucose ≤ 2.2 mmol/L and CSF/blood glucose ratio ≤ 0.45 presented with an earlier onset of symptoms and received an earlier molecular genetic confirm (Table 1).

Absence seizures, paroxysmal exercise-induced dyskinesia, and intellectual disability were significantly associated with a CSF/blood glucose ratio ≤ 0.45 with a relevant impact of sexual differences (Table 1).

The relationships between CSF parameters and developmental functions other than intelligent quotient were scarcely explorable because of the paucity of available data (Table 1; Supplement 1).

Positive response to ketogenic diet was more commonly reported in patients with CSF glucose ≤ 2.2 mmol/L. The same group had a significant response also to antiseizure medications but not to combined treatment (antiseizure medication + ketogenic diet) (Table 1). A significant response to antiseizure medications but not to ketogenic diet was observed in patients with CSF lactate higher than 1 mmol/L (Table 1).

## Discussion

This systematic review suggested a relationship between the levels of CSF biomarkers and the distribution of some clinical parameters (age at the onset of symptoms, seizure and movement disorder types, cognitive profiles, and efficacy of available treatments) in patients with GLUT1 deficiency syndrome (Table 1).

The reliability of hypoglycorrhachia as diagnostic biomarker has been acquired since the last decade, when a retrospective analysis on 147 patients evidenced that CSF was below the 10th percentile of reference range in all published patients while CSF blood/glucose ratio was normal in 10% and data about CSF lactate were often not informative [141]. It was also evidenced that CSF glucose may range between 0.9 to 2.8 mmol/L in GLUT1 deficiency, and milder phenotypes may have CSF values between 2.2 and 2.9 mmol/L [1, 141]. Low CSF glucose mirrors the impoverishment of neuronal bioenergetic resources and might be better correlated with more severe phenotypes [142]. The herein reported data confirmed that hypoglycorrhachia was associated with factors having a relevant therapeutic and prognostic impact (e.g., earlier onset of symptoms and efficacy of ketogenic diet and/or antiseizure medications) (Table 1). The negative impact of hypoglycorrhachia on the developing brain is probably independent by etiologies even if the severity of its effects may vary in the different age ranges [143–146]. The concept of “window of vulnerability” to the damages, induced by the low quote of glucose available to face the increased energy demands by the immature brain in the first months of life, might explain the worst outcome observed in late-treated patients with hypoglycorrhachia and earlier onset of symptoms [2, 108]. This window was placed by some authors between the first and the sixth month after birth and overlapped with the ideal timing for the beginning of ketogenic diet and the optimization of the related therapeutic results [2, 75].

CSF blood/glucose ratio may be considered an index of the degree of transporter function impairment with a higher sensitivity towards the detection of later onset and less severe phenotypes in which a residual activity of GLUT1 might be preserved [142]. The significant association of CSF/blood glucose ratio ≤ 0.45 with sex differences that was observed in published patients has no clear explanations (Table 1). No evident sex predisposition to GLUT1 deficiency was highlighted in the literature even if a negative regulation of GLUT1 protein mediated by estrogens has been reported in in vitro models [1, 147].

The association between low CSF/blood glucose ratio and absence seizures supported the results of previous studies that had highlighted the etiological role of SLC2A1 pathogenic variants in a proportion of cases ranging between 5.6% and 10% of all patients presenting with a childhood onset absence epilepsy [37, 105, 148]. Other authors had evidenced the same pathogenic role in a lower percentage of cases (1.4%) if the analyzed cohorts included patients with all types of idiopathic generalized epileptic syndromes [37, 105, 149]. In these cases, the indication for a lumbar puncture should be mandatory for children in which epilepsy is associated with other clinical hallmarks such as the predominance of seizures after periods of fasting, developmental impairment, or paroxysmal exercise-induced dyskinesia [105]. The lack of significance for seizure types other than absences might contraindicate the procedure especially if epilepsy is the only clinical manifestation [105, 149].

The pathomechanisms behind the higher frequency of early onset absences among patients with a lower CSF/blood/glucose ratio might result from a lower functional adaptability to energy deprivation of mutated neuronal transporters in the networks involving thalamus and connected cortical areas including posterior cingulate cortex, precuneus, angular gyrus, supramarginal gyrus, parietal superior, and occipital mid-region [37, 78, 105, 148, 149] (Table 1). Similar mechanisms might be involved in the impairment of astrocyte-to-neuron lactate shuttles in putamen and cortico-striatal pathways in patients with paroxysmal exercise-induced dyskinesia [78].

The assessed links between CSF/blood glucose ratio and intellectual disability are probably due to the lower ability of impaired cerebral GLUT1 to face the higher functional request of developing brain, and it was firstly highlighted in a nationwide survey including 33 Japanese patients carrying missense variants of SLC2A1 [71]. A comparable correlation between CSF/blood glucose ratio and severity of speech and language impairment was not demonstrated in a smaller Italian series of eight patients [127].

Data from the analyzed literature did not demonstrate any association between lactate levels and developmental/clinical hallmarks of GLUT1 deficiency (Table 1) despite various basic/science and clinical studies highlighted the role of lactate in various cerebral networks, especially the ones involved in learning and memory [142, 150]. A better response to antiseizure medications was evidenced in patients with normal levels of lactate suggesting a less-compromised epilepsy phenotype in patients with a better basal energy metabolic compensation (Table 1).

The maintenance of lumbar puncture in the early diagnostic work-up of patients with GLUT1 deficiency syndrome, despite the increased quote of rapid turnaround time for genetic testing, has relevant prognostic implications because it still allows an earlier access to an effective disease-modifying treatment such as ketogenic diet. The herein reported data highlighted a more significant response to ketogenic in patients with CSF glucose ≤ 2.2 mmol/L while significance was not relevant in patients with low CSF/blood ratio (Table 1). Conversely, another recent systematic review of 230 published patients confirmed significant improvement in movement disorders in 104 out of 127 patients with better results in children with higher CSF/blood glucose ratio [3]. Another direct relationship between CSF/blood glucose ratio and total and verbal IQ improvement, after the introduction of ketogenic diet, was found in a smaller single center series of 14 patients [75].

Few correlations between genotypes and CSF glucose and CSF/blood glucose ratio may be highlighted. Variants c.997C>T (p.Arg333Trp) and c.457C>T (p.Arg153Cys) result in a less flexible and efficient glucose transport because of a higher quote of salt bridges stabilizing the conformation of GLUT1 protein and reducing electrostatic interactions during the activation of its intracellular and transmembrane segments [151]. The variant c.884C>T (p.Thr295Met) results in conformational changes that inhibit the access of glucose molecules in the exofacial site of the transporter [152].

Some limitations, mainly due to the predominant retrospective nature of the collected data, should be considered in the interpretation of the results of this systematic review: (a) differences in the ages of clinical evaluation, cerebrospinal fluid acquisition, achievement of molecular genetic confirm, and introduction of ketogenic diet or antiseizure medications; (b) lack of CSF measurements for a considerable number of published patients; and (c) differences in the methodologies used for the definition of phenotypic severity, clinical variability, response to treatments, and developmental outcome.

## Conclusions

The analysis of the literature confirmed the usefulness of lumbar puncture for the early identification of patients with severe and mild phenotypes of GLUT1 deficiency and the subsequent prognostic implications of an earlier initiation of ketogenic diet. CSF analysis is easily and diffusively available diagnostic tool and contributes to a faster diagnostic work-up in patients with more severe phenotypes. The turnaround time of ultra-rapid next-generation sequencing techniques will not probably impact on the real-world therapeutic planning for the next few years. The results of this systematic review highlighted that a lumbar puncture should be strongly considered when severe developmental delay, paroxysmal exercise-induced dyskinesia, and early onset absences co-exist.

### Supplementary Information

Below is the link to the electronic supplementary material.

Supplementary file1 (XLSX 138 KB)

Supplementary file2 (XLSX 41 KB)

Supplementary file3 (XLSX 86 KB)

Supplementary file4 (XLSX 99 KB)

Supplementary file5 (PNG 144 KB)

Supplementary file6 (DOCX 32 KB)

## Abbreviations

CSF: Cerebrospinal fluid
GLUT1: Glucose transporter 1
EEG: Electroencephalogram
FDG PET: 18-F-fluorodeoxyglucose positron emission tomography
IQ: Intelligence quotient
SLC2A1: Solute carrier family 2 member 1
ADHD: Attention deficit hyperactivity disorder
F: Female
ID: Intellectual disability
IQ: Intelligent quotient
M: Male
N: No
NA: Not available
Y: Yes
M: Mean
SD: Standard deviation
PED: Paroxysmal exercise‐induced dyskinesia
GDD: Global developmental delay
ID: Intellectual disability
