# Supplementary figures and images for: The diagnostic and prognostic role of cerebrospinal fluid biomarkers in glucose transporter 1 deficiency: a systematic review
Source: Eur J Pediatr. 2024 Jul 2;183(9):3665–78. doi: 10.1007/s00431-024-05657-6 (PMC11322378; doi:10.1007/s00431-024-05657-6)

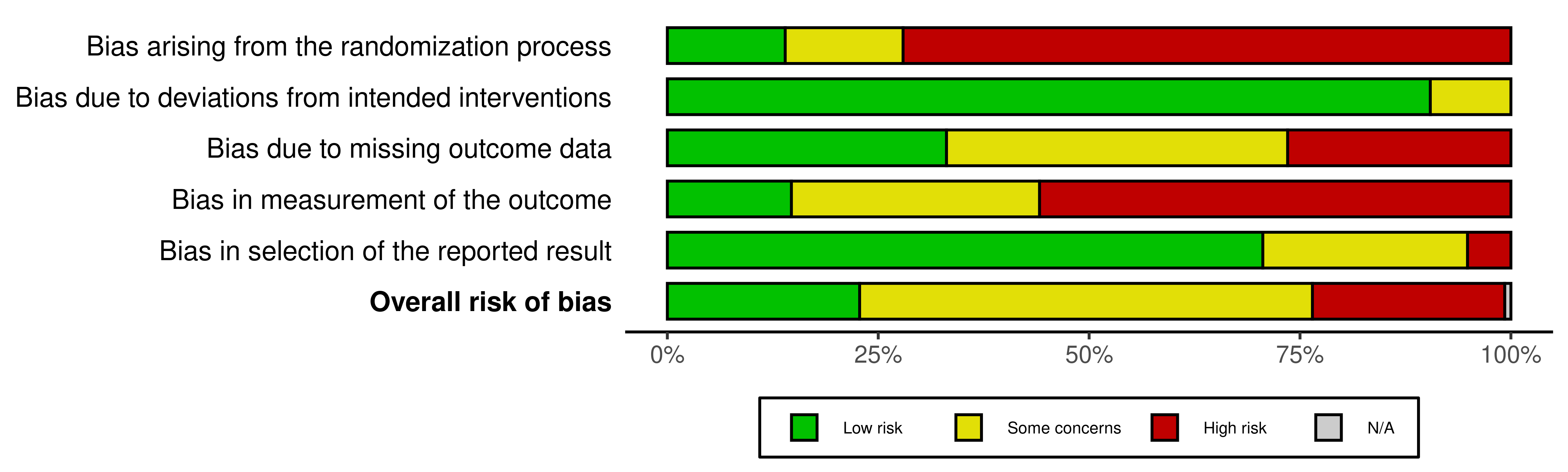

Supplement: Supplementary file 5 — Supplementary file5 (PNG 144 KB) [file 431_2024_5657_MOESM5_ESM.png]
